# Supplementary material for: Aquaporins Respond to Chilling in the Phloem by Altering Protein and mRNA Expression
Source: Cells. 2019 Feb 27;8(3):202. doi: 10.3390/cells8030202 (PMC6468725; doi:10.3390/cells8030202)
Supplement: Supplementary file 1 [file cells-08-00202-s001.pdf]

Supplementary Materials: The following are available online at www.mdpi.com/xxx/s1, Supplementary Table S1: Primer sequences used for the gene expression study.

		Amplicons		
		Forward Primer (5′→3′)	Reverse Primer (5′→3′)	Reference
PtPIP1;1	POPTR_0010s 19930	GCTCGTAGTCTTGGTGCAGCTATC	AATGGGCCAACCCAGAAGATCCAG	this paper
PtPIP1;4	POPTR_0006s 09920	ACTTGGCGTTGAGATTCTTGGC	ATAGGTAGTGGTGCCAGGACAG	this paper
PtPIP2;4	POPTR_0008s 03950	GGACTCCCATGTGCCTGTATTG	AGGGTTGATGCCAGTTCCTGTG	this paper
PtPIP2;5	POPTR_0006s 12980	AAGGGACTCCCATGTTCCTGTG	TGAACCATGAAGACAGCAAATCCG	this paper
PtPIP2;8	POPTR_0009s 01940	ACTGCTGGTATCTCTGGAGGAC	TCCTCGCCAGAAACAGACCAAAG	this paper
PtaSUT4	POPTR_0002s 10710	ATCCTTGGGACTTGGACAAGGGTT	TGATCGAGGAATACYCAAGATGGC	[51]
EF1B	POPTR_0001s 23190	AAGAGGACAAGAAGGCAGCA	CTAACCGCCTTCTCCAACAC	[51]
